# Nephroptosis and ureteroinguinal incarcerated hernia mimicking acute appendicitis

**DOI:** 10.1186/s12894-024-01549-x

**Published:** 2024-07-29

**Authors:** Michal Gergel, Ivan Brychta, Anita Lancz-Klikacova, Alexander Mayer

**Affiliations:** 1 University Hospital Bratislava, Bratislava, Slovakia; 2 Slovak Medical University, Bratislava, Slovakia

**Keywords:** Ureteroinguinal hernia, Acute appendicitis, Ureteral incarceration

## Abstract

The involvement of kidney, perirenal fat, and ureter is a rare variant of inguinal hernia. We report a case of a 78-year-old man presenting with typical clinical signs of acute appendicitis. Ultrasonography and CT scan revealed ptosis of the right kidney with a major part of the perirenal capsule involved in a large right sided inguinal hernia with acute obstruction of the ureter and urostasis. Acute surgery was performed, involving resection of perirenal fat, liberation, resection, and neoimplantation of the ureter, and hernioplasty. The postoperative period was uneventful. This case illustrates diagnostic unpredictability of acute appendicitis as well as anatomic variety of inguinal hernias.

## Introduction

The inguinal hernia and its complications represent a common condition routinely treated by general surgeons worldwide; however, a large spectrum of clinical and anatomical variabilities can turn a routine inguinal hernia into a surgical challenge. Ureteroinguinal hernias in patients with native kidneys are rare with only approximately 180 cases reported so far [1–3]. The involvement of the ureter in an inguinal hernia can be a surprise finding during a planned hernia surgery with a high risk of recognized or unrecognized ureteral injury. Other cases were diagnosed following the differential diagnosis of obstructive uropathy or obstructive pyelonephritis. The herniation of perirenal fat may be associated with ureteral hernias, as well [4]. A specific form of ureteroinguinal hernia involves the kidney – either ectopic or ptotic. The complete herniation and incarceration of the kidney in an inguinal hernia has been described as an extremely rare finding [5, 6].

Pyelonephritis of an ectopic or ptotic right kidney has also been described as a cause of right lower quadrant pain, making it an extremely rare differential diagnosis when considering acute appendicitis [7–9]. Imaging by ultrasonography and/or CT would reveal this unexpected condition with almost absolute accuracy, but these imaging techniques are not routinely utilized, either because of clear clinical findings or because of their unavailability. The combination of ureteroinguinal hernia with an acute incarceration of the ureter with ptotic right kidney mimicking acute appendicitis has, to our knowledge, not yet been described.

## Case report

A 78-year-old Caucasian male treated chronically for arterial hypertension and neuropathy, with a past surgical history of bilateral hip arthroplasty, presented to the emergency department with acute abdominal pain. The pain began the previous day and worsened gradually over 32 h, starting as diffuse abdominal pain that migrated to the right lower quadrant with radiation to the lumbar region. Other than anorexia, no gastrointestinal symptoms were present. The patient had a well-known right-sided inguinal hernia that was managed conservatively as the patient refused surgical intervention. Current subjective complains were not associated with any local changes nor pain related to the hernia.

The patient´s body measures were 175 cm and 85 kg (BMI 27.7 kg/m2) Physical examination showed painful tenderness the right lower quadrant. Palpation of the McBurney point (Bloomberg sign, ) as well as percussion in the right lower quadrant (Pleniés sign) were painful. Palpation of the left side (Rovsing sign) was painless and rectal examination was negative. There was a right sided inguinal hernia with a large irreducible sac reaching deep into scrotum measuring 15 × 7 cm. The sac was slightly painful; however, typical signs of incarceration were absent.

**Fig. 1:**
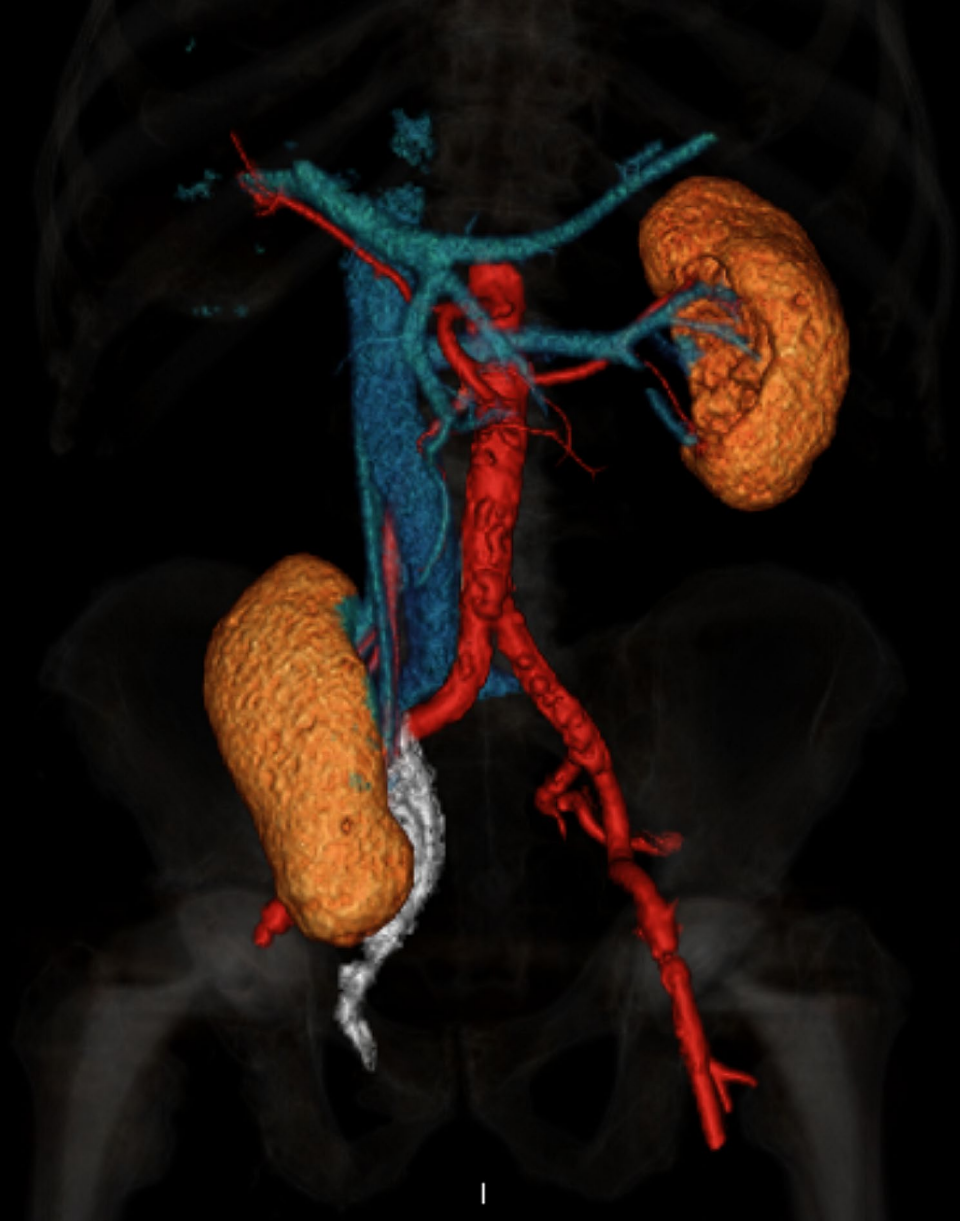
3D reconstruction of CT scan, showing ptotic right kidney with normal origin of renal vessels and ureter obstructed in inguinal ring

**Fig. 2:**
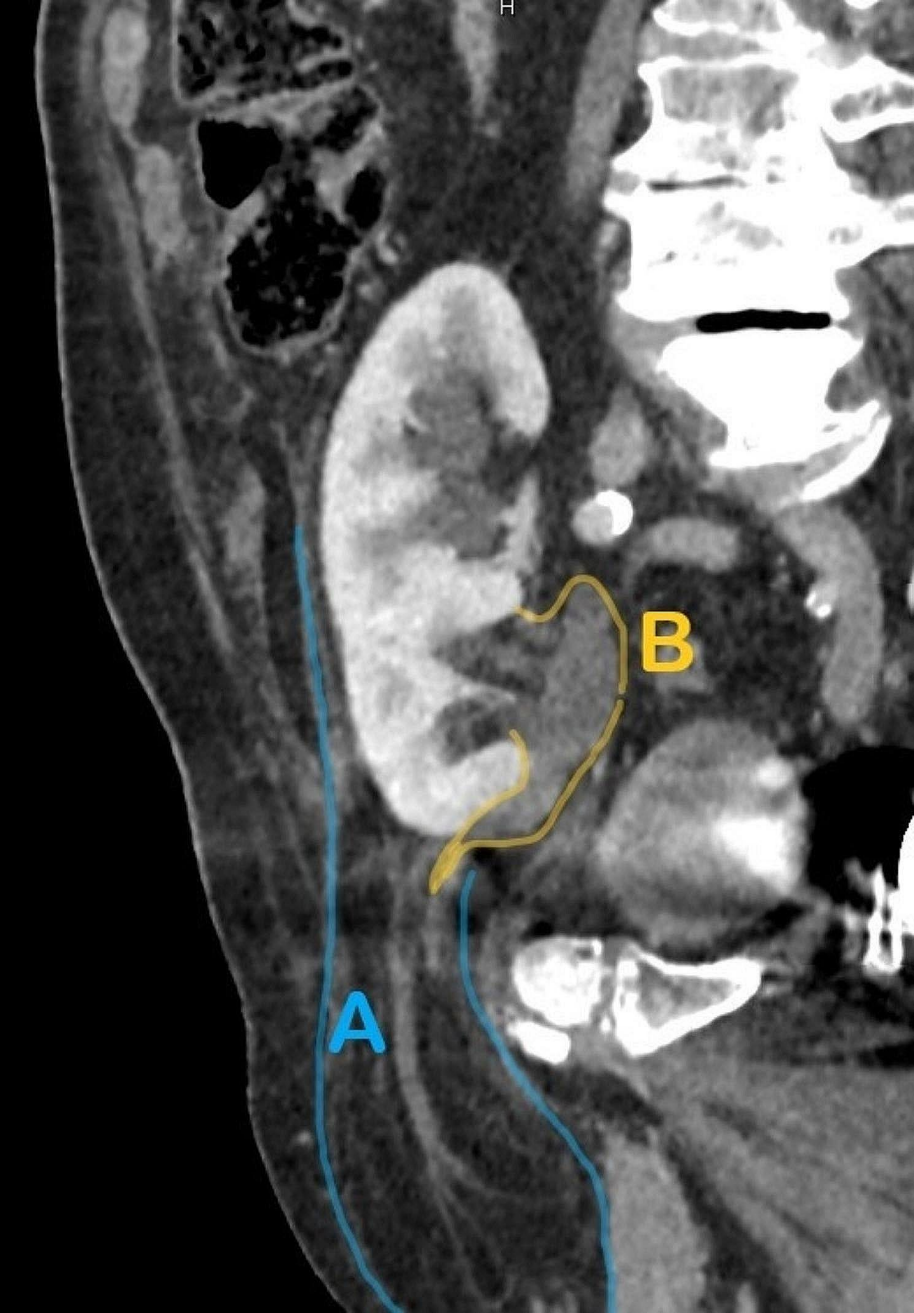
CT scan showing herniated perirenal fat (**A**) and dilated ureter with obstruction in the internal inguinal ring (**B**)

**Fig. 3:**
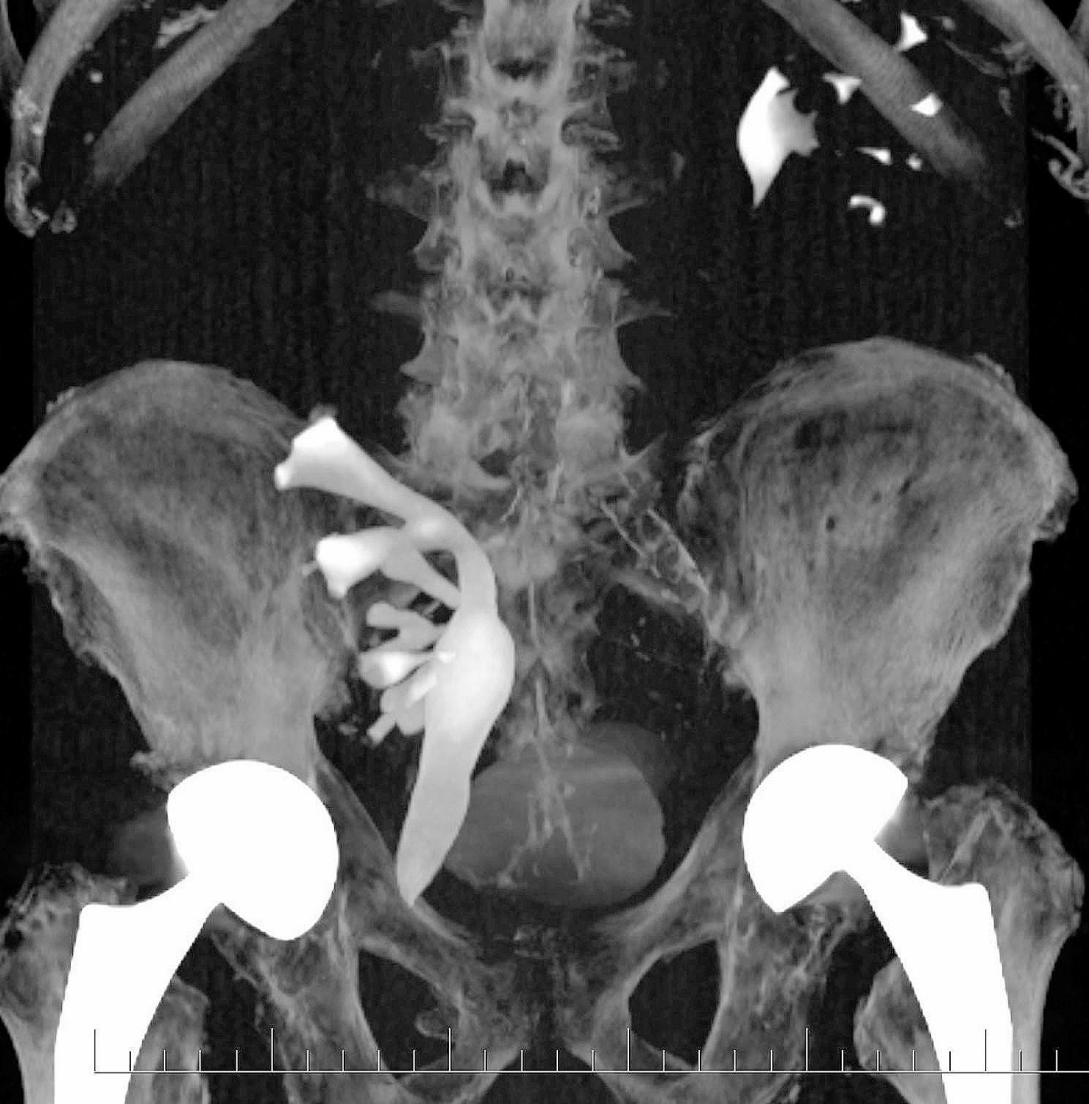
Reconstructed CT urography showing normal position of the left kodney, and ectopic position of the right kidney with urostasis

Laboratory findings showed significant inflammatory activity: CRP 293 mg/l, leucocyte count 21,500/μl. Our first line diagnosis, considering typical current complaints and clinical findings, was acute appendicitis. Nevertheless, we considered complications related to the large inguinal hernia, as well. As a result, we requested abdominal ultrasonography. Surprisingly, it showed a medially located cecum with no signs of appendicitis and an ectopic right kidney located in the right hypogastrium with slight dilation of the pelvis, calices, and proximal ureter. The point of maximal pain was clearly located at the kidney. Following this unexpected finding, we performed a CT scan, which showed the right ptotic kidney located in the right iliac fossa, with grade 2 to 3 renal stasis. (Fig. 1)(Fig. 2)(Fig. 3) The renal artery and vein were stretched from their normal anatomic origin caudally towards the descended kidney hilum. Renal perfusion in both arterial and venous phases of the scan was normal. The right renal capsule was almost completely dislocated in the inguinal hernia. The ureter was proximally dilated to 15 mm and was headed towards the inguinal hernia with apparent constriction in the hernia neck. Considering these findings, we concluded the final diagnosis of a sliding right inguinal hernia containing the capsule of the right kidney with acute obstruction (incarceration) of the right ureter. Painful palpation of the right kidney along with elevated inflammatory markers strongly indicated acute obstructive pyelonephritis, although urinalysis showed completely negative results. Due to the risk of ischemic damage to the right kidney and the ureter, as well as risk of urosepsis, we initiated empiric antibiotic therapy with ceftizoxime and recommended urgent surgery.

**Fig. 4:**
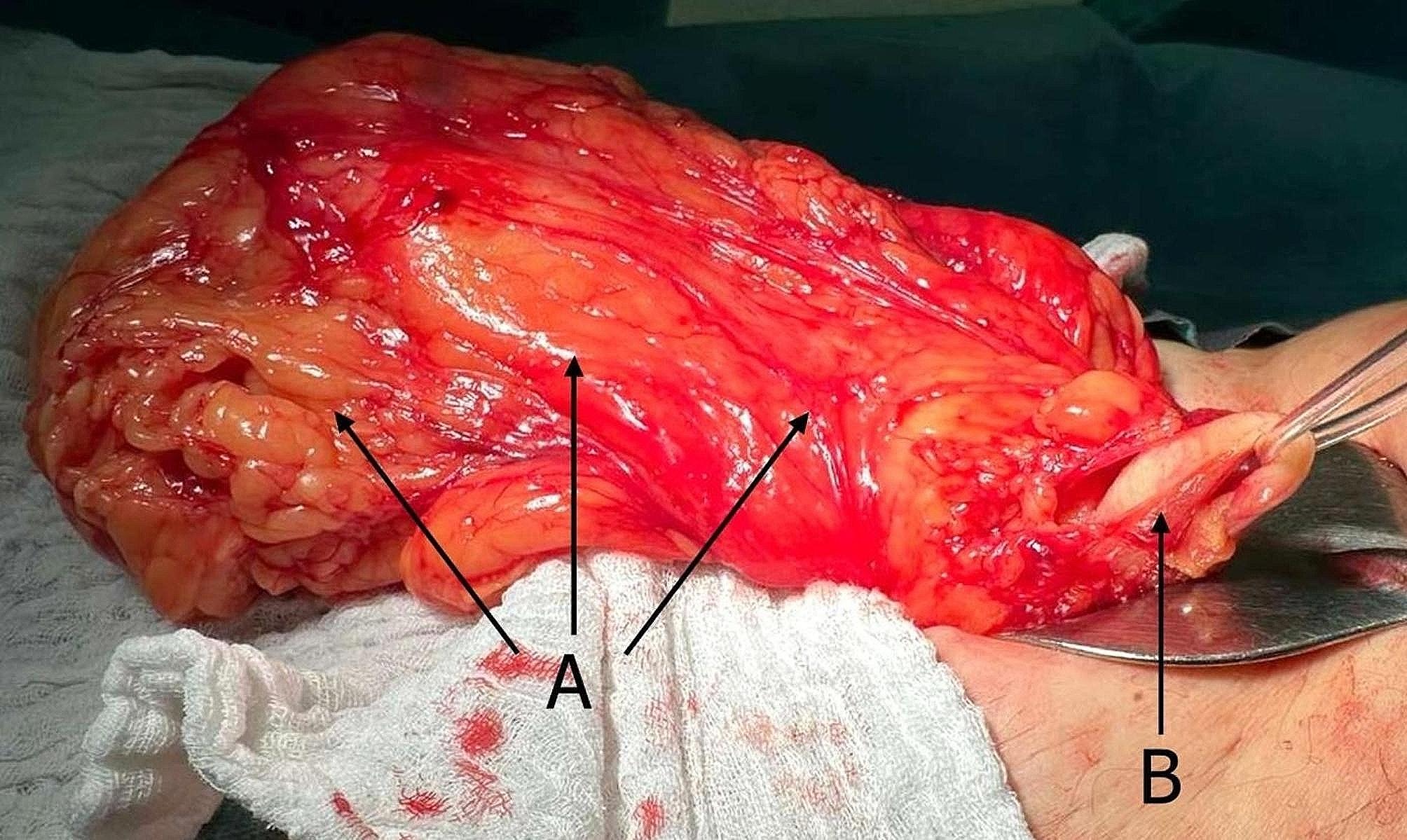
Inguinal hernia containing perirenal fat (**A**) and dilated ureter (**B**)

**Fig. 5:**
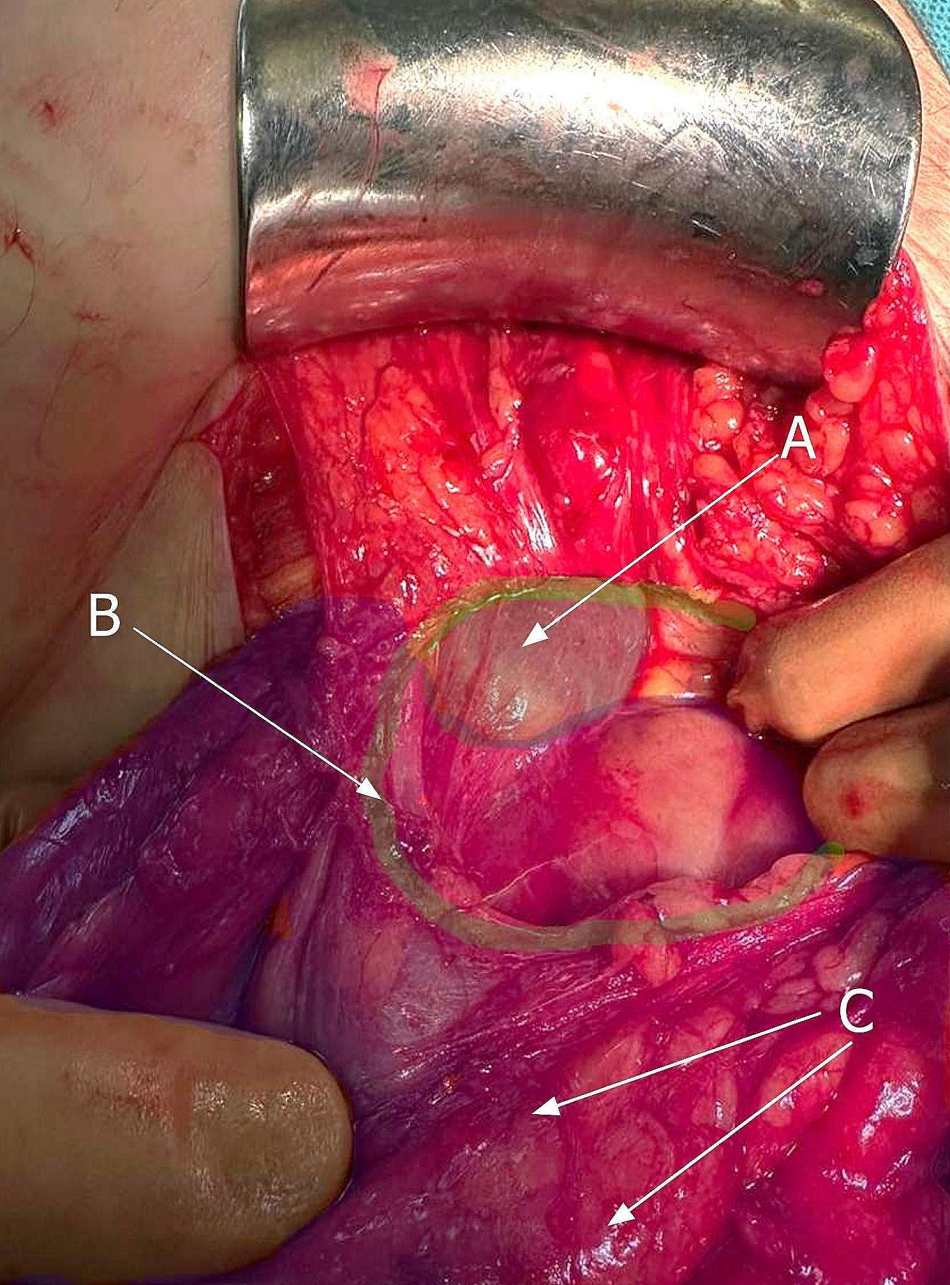
Inferior pole of the right kidney (**A**) visible in the internal inguinal ring (**B**) with perirenal fat (**C**) involved in the inguinal hernia

Surgery was performed under general anesthesia. A Foley urinary catheter was inserted, and the urinary bladder was filled with 50 ml normal saline. Through a standard inguinal incision, the external ring was visualized, and the hernia content was extracted from the right hemiscrotum revealing well bordered fatty tissue with a fibrous capsule. After the incision of the anterior wall of the inguinal channel, we identified an epigastric artery located medial to the hernia neck, confirming an anatomically indirect hernia. The spermatic cord was identified dorso-medially to the neck and isolated. Throughout the preparation of the hernia content, no peritoneal structures were involved. The ureter was located in the medial proximal part of the hernia and was pulled to the external orifice with significant dilatation of the proximal half. There were no signs of ischemia, and shortly after liberation, typical ureteral peristalsis was present. After the incision of the frontal wall of the fibroadipose tissue in the external inguinal ring, the inferior pole of the right kidney was revealed as part of the content of the hernia neck heading towards the hernia. Despite this, there were no signs of mechanical damage to the visible part of the kidney. After the complete separation of the ureter and manual repositioning of the kidney cranially, no other anatomical structures were found in the hernia neck. Almost the whole perirenal fat capsule, which was contained in the hernia, was completely resected at the level of inferior renal pole. (Fig. 4)(Fig. 5) The whole formation measured 23 × 10 × 6 cm and weighed 850 g.

The right ureter was approximately 30 cm long, and, as the kidney was dislocated in the right iliac fossa, the direct distance from the inferior pole to the urinary bladder was reduced to approximately 5 cm. The ureter was, therefore, extremely crooked and prone to future kinking. As a result, we resected a 10 cm segment, ligated the distal part, and implanted the proximal segment to the bladder wall by direct, single layer continuous suture, with a JJ stent (16 cm, 7 French). A Redon drain was placed in the space of Retzius to control possible urinary leak. Finally, we performed a Liechtenstein hernioplasty using polypropylene mesh 10 × 15 cm for definitive treatment of the inguinal hernia.

Perioperative urine sample from the ureter showed sterile urine. The postoperative period was uneventful, apart from mild hematuria, with satisfactory reduction of inflammatory parameters. Renal parameters showed no significant pathology. The urinary catheter was removed after 4 days, and the patient was discharged for outpatient treatment. The JJ stent was removed 6 weeks after the procedure.

## Discussion

The clinical symptoms of our patient were typical for acute appendicitis with no complaints of hernia changes or dysuria. Physical examination showed no specific findings of incarceration, and the kidney was not palpable due to central obesity. Many experienced surgeons rely on clinical findings to diagnose acute appendicitis, subsequently referring these patients for appendectomy. Although we do not consider ultrasonography a necessary measure, we request it in all clinically unclear cases, as well as in elderly patients. Our case suggests that ultrasonography should be considered a routine method if it is available.

The CT scan showed obstruction of the right ureter and acute hydronephrosis. Acute obstructive pyelonephritis was very likely, considering significant elevation of inflammatory markers. We considered less invasive options, such as ureteral stenting and percutaneous nephrostomy. Ureteral perturbation was likely to fail due to kinking of the ureter. While nephrostomy was easily achievable, only surgery was capable of providing definitive treatment.

The normal anatomical position of the kidney is stabilized by the perirenal fat and fascias. The sliding of this fat capsule towards the inguinal channel and further to the inguinal hernia caused instability of the kidney position and its descent into the right iliac fossa and further towards the inguinal channel. The lack of any specific symptoms, apart from the apparent inguinal hernia, suggests that the process of descent was gradual.

The right kidney was thoroughly examined with no signs of ischemia or other pathology. Resection of perirenal fat, shortening of the ureter, and hernia repair appear to be sufficient measures to stabilize the position of the kidney and maintain its full function.

Liberating the ureter from the hernia may be sufficient for the treatment of urinary obstruction; however, significant descent of the kidney increased risk of future ureteral kinking. At this point, resection of the ureter and neoimplantation was considered to be the best option.

## Conclusion

An inguinoscrotal hernia involving the perirenal fat capsule and ureter (ureteroinguinal hernia) is a rare condition with variable clinical presentations. It is associated with nephroptosis and may cause acute kidney obstruction and possible obstructive pyelonephritis. The clinical presentation in such a situation can persuasively mimic acute appendicitis. Ultrasonography and/or CT may not necessarily be performed in clinically clear cases of acute appendicitis; however, it is highly advisable to request further imaging when atypical local changes, e.g. inguinal hernia, are present.

## Data Availability

All original data are included in patient´s medical records and internal medical database of University Hospital Bratislava. All data generated or analysed during this study are included in this published article.

## Abbreviations

BMI: Body mass index
CT: Computer tomography
US: Ultrasound
